# Elevated Serum Levels of IGF-1 Are Sufficient to Establish Normal Body Size and Skeletal Properties Even in the Absence of Tissue IGF-1

**DOI:** 10.1002/jbmr.20

**Published:** 2010-01-14

**Authors:** Sebastien Elis, Hayden-William Courtland, Yingjie Wu, Clifford J Rosen, Hui Sun, Karl J Jepsen, Robert J Majeska, Shoshana Yakar

**Affiliations:** 1 Division of Endocrinology, Diabetes and Bone Disease, Mount Sinai School of MedicineNew York, NY, USA; 2 Maine Medical Center Research InstituteScarborough, ME, USA; 3 Leni and Peter W May Department of Orthopaedics, Mount Sinai School of MedicineNew York, NY, USA; 4 Department of Biomedical Engineering, City College of New YorkNew York, NY, USA

**Keywords:** IGF-1, bone, transgenic mice, IGF-1KO, micro-computed tomography, endocrine IGF-1, mechanical properties

## Abstract

Use of recombinant insulin-like growth factor 1 (IGF-1) as a treatment for primary IGF-1 deficiency in children has become increasingly common. When untreated, primary IGF-1 deficiency may lead to a range of metabolic disorders, including lipid abnormalities, insulin resistance, and decreased bone density. To date, results of this therapy are considered encouraging; however, our understanding of the role played by IGF-1 during development remains limited. Studies on long-term treatment with recombinant IGF-1 in both children and animals are few. Here, we used two novel transgenic mouse strains to test the long-term effects of elevated circulating IGF-1 on body size and skeletal development. Overexpression of the rat igf1 transgene in livers of mice with otherwise normal IGF-1 expression (HIT mice) resulted in approximately threefold increases in serum IGF-1 levels throughout growth, as well as greater body mass and enhanced skeletal size, architecture, and mechanical properties. When the igf1 transgene was overexpressed in livers of igf1 null mice (KO-HIT), the comparably elevated serum IGF-1 failed to overcome growth and skeletal deficiencies during neonatal and early postnatal growth. However, between 4 and 16 weeks of age, increased serum IGF-1 fully compensated for the absence of locally produced IGF-1 because body weights and lengths of KO-HIT mice became comparable with controls. Furthermore, micro-computed tomography (µCT) analysis revealed that early deficits in skeletal structure of KO-HIT mice were restored to control levels by adulthood. Our data indicate that in the absence of tissue igf1 gene expression, maintaining long-term elevations in serum IGF-1 is sufficient to establish normal body size, body composition, and both skeletal architecture and mechanical function. © 2010 American Society for Bone and Mineral Research.

## Introduction

The growth hormone (GH)–insulin-like growth factor 1 (IGF-1) axis is notably complex owing to the number of IGF-1-binding proteins that modulate its activities,(1) the ability of GH and IGF-1 to act individually as well as collectively, and in particular, the ability of IGF-1 to act via both endocrine and autocrine/paracrine pathways.(2–4) As an endocrine agent, IGF-1 is the major circulating hormone produced by the liver, principally in response to GH; as a paracrine factor, IGF-1 is produced by a wide range of cell types, where its expression is regulated not only by GH but also by numerous other local and systemic agents.

This complexity of the GH–IGF-1 system is especially evident in its control of skeletal development. Both igf1 null and GH receptor (ghr) null mice showed reduced bone length, total cross-sectional bone area, and cortical bone area compared with wild-type mice,(5,6) but igf1 null mice exhibited greater impairment in bone accretion than mice lacking GHR or GH, suggesting GH-independent IGF-1 effects during postnatal growth. Further, mice lacking GHR activation (eg, Snell dwarf, Ames dwarf, or ghr null mice) are indistinguishable from their control littermates up to 3 weeks after birth, but then their relative size decreases to 60% to 50% of controls, confirming the important role of GH in postnatal longitudinal growth.(7) On the other hand, the relative size of igf1 null mice is low at birth (60% of controls) and then decreases progressively to 30% of adult control size, revealing the importance of IGF-1 in both pre- and postnatal growth.(8,9)

IGF-1 regulates the growing skeleton via both endocrine and autocrine/paracrine pathways. In liver-IGF-1-deficient (LID) mice, which Exhibit 75% reductions in serum IGF-1 but normal skeletal IGF-1 expression, serum IGF-1 regulates periosteal bone growth and determines bone size and bone strength.(10) Moreover, we found that decreases in endocrine (serum) IGF-1 levels impair skeletal development only at the onset of puberty (beginning after 4 weeks of age, at which time IGF-1 normally peaks in serum).(10) We therefore proposed that endocrine IGF-1 is a major determinant of skeletal growth at prepubertal age and during puberty, whereas autocrine/paracrine IGF-1 regulates skeletal growth early pre- and postnatally (up to 4 weeks).

During the last decade, it became evident that administration of recombinant IGF-1 to GH-resistant patients (Laron's dwarfs)(11–15) or to ghr null mice(16) results in increased longitudinal growth, suggesting that elevated levels of endocrine IGF-1 can stimulate growth of the prepubertal and adult skeleton, even in states of diminished autocrine/paracrine IGF-1 action. Here, we sought to determine whether increased levels of endocrine IGF-1 can rescue the severe skeletal phenotype of igf1 null mice and at what time during development this may occur. We performed longitudinal analyses of three mouse models: (1) control mice, which express normal levels of tissue and serum IGF-1, (2) hepatic igf1 transgenic (HIT) mice, which express normal levels of tissue IGF-1 but overexpress the rat igf1 transgene in liver and therefore have increased serum IGF-1 levels, and (3) mice with the igf1 gene totally ablated but overexpressing the rat igf1 transgene in liver (KO-HIT), therefore exhibiting increased serum IGF-1 levels in the absence of igf1 gene expression in tissues.(17)

We found that increased serum IGF-1 levels in HIT mice led to increased body size and enhancement of skeletal morphologic parameters and skeletal strength. In contrast, autocrine/paracrine IGF-1 deficiency in KO-HIT mice hindered early postnatal skeletal growth despite elevated levels of serum IGF-1. Nonetheless, by adulthood, increased serum IGF-1 levels compensated for these deficiencies and even restored skeletal strength above control levels.

## Materials and Methods

### Animals

Female HIT and KO-HIT mice (on FVB/N background) were generated as described previously.(17) All mice were homozygous for the igf1 transgene. Female mice were housed four per cage in a clean mouse facility, fed standard mouse chow (Purina Laboratory Chow 5001, Purina Mills, Gray Summit, MO) and water ad libitum and kept on a 12-hour light/dark cycle. Animal care and maintenance were provided through the Mount Sinai School of Medicine's AAALAC Accredited Animal Facility. All procedures were approved by the Animal Care and Use Committee of the Mount Sinai School of Medicine.

### Serum hormones

Mice were bled through the mandibular vein, and serum samples were collected between 7 and 9 a.m. on a fed state at the indicated ages. Serum IGF-1 and GH levels were determined using commercial radioimmunoassays, as described previously.(18–20)

### Body composition

Body composition (fat and lean mass) was assessed in live (nonanesthetized) animals using MRI (EchoMRI 3-in-1, Echo Medical Systems, LLC, Houston, TX). This technique allows highly precise serial measurements without affecting the subjects tested. The measurement of each mouse lasts 90 seconds, and the precision of the measurement is between 0.1 and 0.3 SD.

### Micro-Computed tomography (µCT)

Cortical bone morphology at the midfemoral diaphysis and trabecular bone volume fraction and microarchitecture in the excised distal femoral metaphysis were assessed as described previously.(10) Femurs were reconstructed at an 8.7-µm voxel resolution. For trabecular bone regions, we assessed the bone volume fraction (BV/TV, %), trabecular thickness (Tb.Th, µm), trabecular number (Tb.N), and trabecular spacing (Tb.Sp, µm). For cortical bone at the femoral midshaft, we measured the average total cross-sectional area inside the periosteal envelope (Tt.Ar, mm^2^), the cortical bone and medullary area within this same envelope (Ct.Ar, mm^2^, and Ma.Ar, mm^2^, respectively), the relative cortical area (RCA, Ct.Ar/Tt.Ar, %), the average cortical thickness (Ct.Th, µm), and the polar moment of inertia (J_o_). Robustness was defined as Tt.Ar/Le; a higher ratio denotes more robust bone. All regions of analysis were standardized according to anatomic landmarks. Tissue mineral density (TMD) was defined as the average mineral value of the bone voxels and expressed in hydroxyapatite density equivalents (HA mg/cm^3^).

### Mechanical testing

Mouse femurs from 8- and 16-week-old control, HIT, and KO-HIT mice were tested to failure by four-point bending using a servohydraulic materials testing system (Instron Corp., Canton, MA, USA). This test measures whole-bone stiffness, maximum load, postyield deflection, and work to failure. Femurs were placed with the anterior surface down on two lower supports. The two lower and two upper supports were set apart by 6.35 and 2.2 mm, respectively. Loading was centered over the midshaft at a displacement of 0.05 mm/s until failure. All mechanical properties were calculated from the load-displacement curves, as described previously.(21)

### Histomorphometry

Eight-week-old animals were injected with calcein (15 mg/kg) 7 and 2 days prior to euthanasia. Femurs were fixed in 10% neutral buffered formalin, embedded in polymethyl methacrylate (PMMA), and sectioned (200 µm thickness) at the middiaphysis using a low-speed diamond-coated wafering saw (Leica, Bannockburn, IL, USA). Sections were adhered to either glass or acrylic slides using a nonfluorescing mounting medium. Final section thicknesses after polishing were between 30 and 40 µm. For determination of osteoclast number, TRAP staining was performed by incubating slides in a TRAP staining solution (0.05 M of sodium acetate, 0.025 M of sodium tartrate, 0.125 mg/mL fast red violet LB salt, and 0.125 mg/mL naphtol AS-MX phosphate) for 180 minutes and then washing with water. For determination of osteoblast number, toluidine blue staining was performed by incubating slides in a solution of 0.02% toluidine blue for 30 minutes and then washing with water. All measurements were performed using an OsteoMeasure System (Osteometrics, Atlanta, GA, USA) in accordance with standard protocols. Sections were imaged using a digital camera attached to a visible light/fluorescence microscope (Zeiss Axioplan2, Zeiss AxioVision, Thornwood, NY, USA).

### Statistical analysis

All bone traits, body weight (BW), body composition, serum hormones, and µCT measurements are presented as means ± SEM. One-way analysis of variance (ANOVA) was used to test for differences among groups at each age (Statview Software Version 5.0, SAS, Institute, Inc., Cary, NC, USA). If ANOVA revealed significant effects, the means were compared by Fisher's test, considering *p* < .05 as significant.

## Results

### Locally produced (autocrine/paracrine) IGF-1 is essential to establish neonatal and early postnatal body size; elevated serum IGF-1 compensates for absence of locally produced IGF-1 only during later postnatal growth

**Fig. 1:**
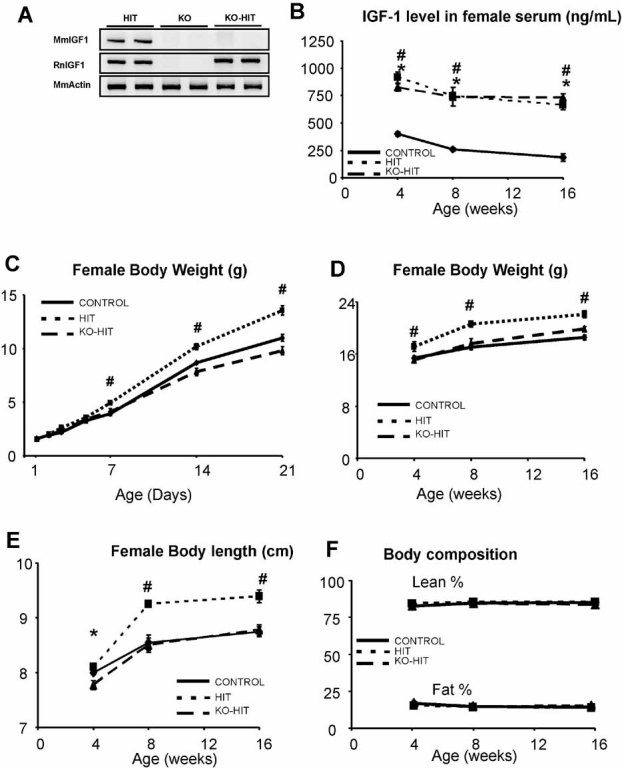
Tissue IGF-1 action is essential for establishment of neonatal and early postnatal body size. The igf1 transgene (RnIGF-1) is expressed in livers (A) of P_0_ HIT and KO-HIT newborns, whereas the endogenous mouse igf1 gene (MmIGF-1) is expressed in HIT mice only. Serum levels of IGF-1 (B) measured from 4 to 16 weeks of age. Body weight measured from 1 to 21 days postnatally (C), body weights from 4 to 16 weeks (D), and length (nose to anus) from 4 to 16 weeks of age (E). (F) Relative lean and body fat mass were determined using NMR as described in “Materials and Methods.” Data are presented as mean ± SEM of n = 10 to 15 mice in each group at each time point. *p < .05 comparing KO-HIT mice with control. ^#^*p* < .05 comparing HIT mice with control.

Female HIT and KO-HIT mice were generated as described previously(17) using hepatocyte-specific transthyretin (TTR) promoter driving the rat igf1 transgene (HIT) (all mice were on FVB/N genetic background, including controls). The TTR promoter is turned on early during embryogenesis,(22–25) and liver RNA isolated from P_0_ newborns showed high levels of expression in both HIT and KO-HIT mice (Fig. 1A). Rat igf1 transgene expression in liver led to threefold increases in serum IGF-1 levels above control mice at 4, 8, and 16 weeks of age in both HIT and KO-HIT mice (Fig. 1B). It is important to note that serum IGF-1 levels did not differ between mice that carry one or two sets of the igf1 transgene, but herein we present only homozygous transgenic mice. GH levels, assessed by ELISA, showed no significant difference among the three groups (control levels ranged from 1.3 to 27.5 ng/mL, HIT 0.8 to 41.2 ng/mL, and KO-HIT 1.1 to 73.0 ng/mL), as shown previously.(17)

Female HIT mice with elevated serum IGF-1 in the presence of normal autocrine/paracrine IGF-1 exhibited greater body weights (Fig. 1C, D) than controls starting at 1 to 2 weeks of age: +17.6% at 2 weeks of age (*p* < .01), +10.2% at 4 weeks of age (*p* < .05), +30.4% at 8 weeks of age (*p* < .0001), and +19.1% at 16 weeks of age (*p* < .0001). In contrast, body weights of KO-HIT mice with elevated serum IGF-1 but no autocrine/paracrine IGF-1 were similar to controls from day 1 to 16 weeks of age (at 8 weeks, *p* = .0624; at 16 weeks, *p* = .1090). Moreover, KO-HIT mice exhibited an approximately twofold increase in body weight compared with igf1 null mice throughout growth.(17) Likewise, elevations in serum IGF-1 resulted in increases in body length in HIT mice relative to control mice at 8 and 16 weeks of age (Fig. 1E). KO-HIT mice, by contrast, were shorter than controls but “caught up” by 8 weeks of age (Fig. 1E). Despite differences in body weight, it is important to note that relative fat and lean mass (normalized to body weights) assessed by MRI did not differ among the groups at any age (Fig. 1F). Together these data suggested that autocrine/paracrine IGF-1, which is absent in KO-HIT mice, plays a critical role in establishing body length prior to 4 weeks of age. Elevated serum IGF-1 levels in neonatal KO-HIT mice failed to overcome this deficiency. On the other hand, between 4 and 8 weeks, increased serum IGF-1 was able to compensate for the absence of locally produced IGF-1 because body lengths of KO-HIT mice became comparable with controls.

### When autocrine/paracrine IGF-1 expression is normal, elevated serum IGF-1 enhances bone structural and mechanical properties

**Fig. 2:**
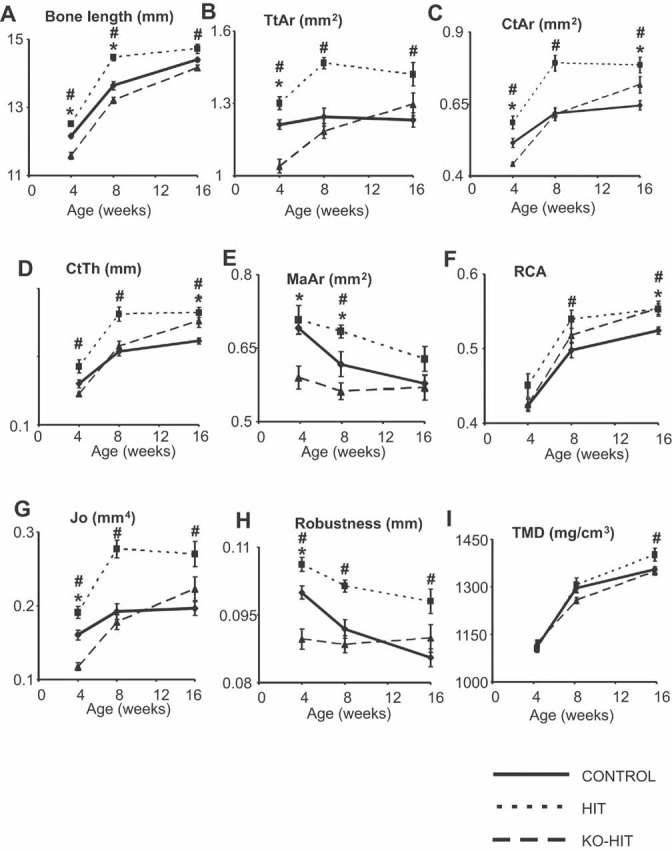
Elevated serum IGF-1 levels restore the cortical envelope architecture in the absence of tissue IGF-1 action. Cortical bone morphology was analyzed at the femur midshaft by µCT. Bone length (A), total cross-sectional area (Tt.Ar) (B), cortical bone area (Ct.Ar) (C), cortical bone thickness (Ct.Th) (D), marrow area (Ma.Ar) (E), relative cortical area (RCA = Ct.Ar/Tt.Ar) (F), polar moment of inertia (G), robustness (Tt.Ar/length) (H), and tissue mineral density (TMD) (I) were measured at 4, 8, and 16 weeks of age. Data are presented as mean ± SEM of n = 10 to 15 mice in each group at each time point. *p < .05 comparing KO-HIT mice with control. ^#^*p* < .05 comparing HIT mice with control.

**Fig. 3:**
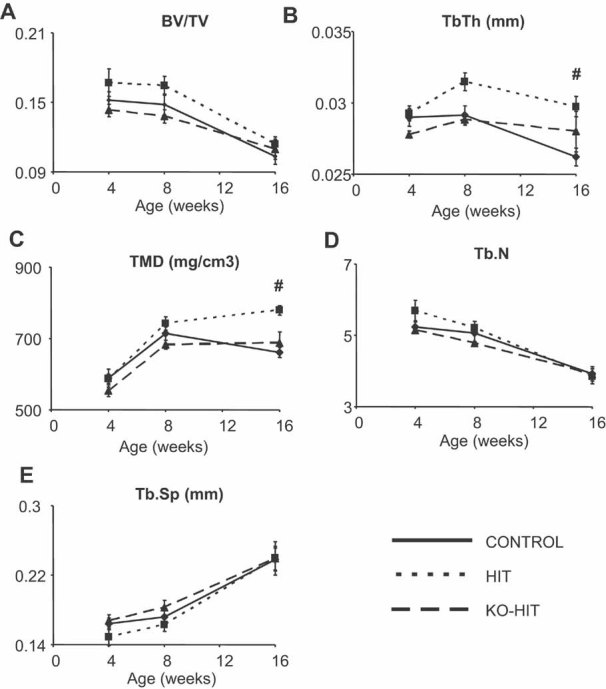
Elevated serum IGF-1 levels are sufficient to preserve cancellous bone in the absence of tissue IGF-1 action. Cancellous bone morphology was analyzed at the distal femur by µCT. Bone volume fraction (BV/TV) (A), trabecular thickness (Tb.Th) (B), tissue mineral density (TMD) (C), trabecular number (Tb.N) (D), and trabecular spacing (Tb.Sp) (E) were measured at 4, 8, and 16 weeks of age. Data are presented as mean ± SEM of n = 10 to 15 mice in each group at each time point. *p < .05 comparing KO-HIT mice with control. ^#^*p* < .05 comparing HIT mice with control.

Female HIT mice with elevated serum IGF-1 showed significant increases above wild-type control mice in a wide range of bone structural features. These included longer femora (2.3% to 4.9%) and greater cross-sectional total area (7.3% to 17.9%), cortical area (13.9% to 25.4%), cortical thickness (11.5% to 17.2%), and polar moment of inertia (19.1% to 42.9%) at all ages (Fig. 2). Cross-sectional areas of HIT femurs also were greater than controls when normalized to bone length (Tt.Ar/Le), indicating that elevated serum IGF-1 produced a more robust (less slender) phenotype (Fig. 2H). Moreover, TMD in HIT mouse femurs was significantly (3.4%, *p* < .01) elevated above control mice at 16 weeks. HIT mice also exhibited enhanced trabecular bone features. µCT analyses of femoral distal metaphyses of 4-, 8-, and 16-week-old female mice revealed that trabecular bone volume/total volume (BV/TV) peaked at 8 weeks of age for all groups and then decreased by approximately 24% to 32% with age (Fig. 3). In HIT mice, trabecular thickness (Tb.Th) was increased over controls by 8% at 8 weeks, whereas both trabecular thickness (13.5%) and TMD (17.9%) were increased at 16 weeks (Fig. 3).

**Fig. 4:**
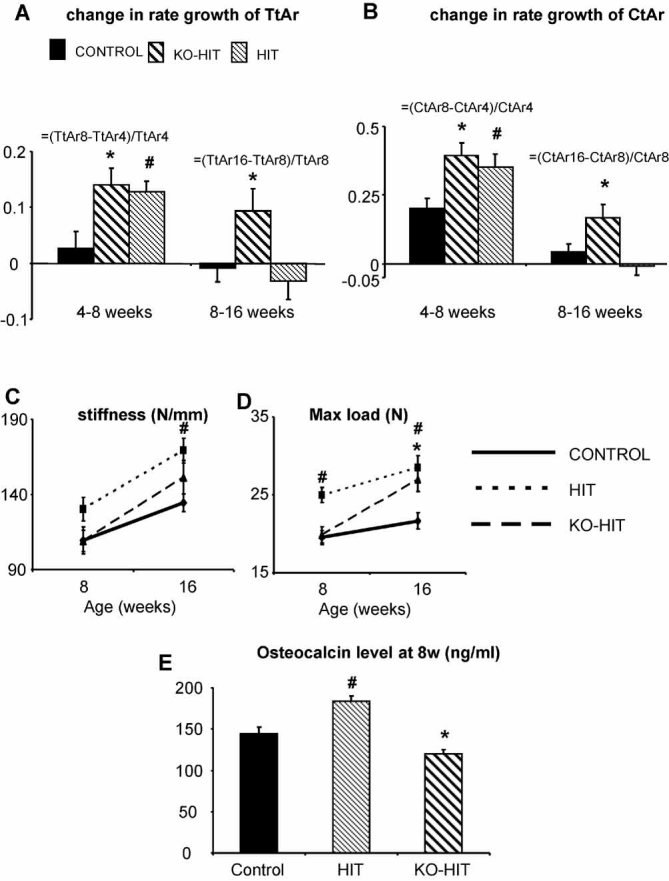
Elevated serum IGF-1 levels restore bone mechanical properties in the absence of tissue IGF-1 action. Mechanical properties were assessed in femurs by four-point bending. Stiffness (A), which indicates resistance to bending, and maximal load (B), which indicates stress at fracture, were measured at 8 and 16 weeks of age. Data are presented as mean ± SEM of n = 10 to 15 mice in each group at each time point. Transversal growth rate indicated by increases in Tt.Ar (C) and Ct.Ar (D) and morphology have been analyzed over two growth periods: from 4 to 8 weeks and from 8 to 16 weeks of age using the following formula: Growth rate of Ct.Ar between 4 and 8 weeks = (CtAr_8w_ – CtAr_4w_)/CtAr_4w_. Data are presented as mean ± SEM of n = 10 to 15 mice in each group over each period of time. Serum osteocalcin levels (E) were measured at 8 weeks of age. *p < .05 comparing KO-HIT mice with control. ^#^*p* < .05 comparing HIT mice with control.

The rapid growth of HIT mice was indicated by an increased transverse growth rate, which was calculated as the increase in total cross-sectional area between 4 to 8 and 8 to 16 weeks of age (Fig. 4A). We observed similar increases in cortical bone area during these two age intervals (Fig. 4B). Consequently, the enhanced morphologic and compositional phenotypes of HIT mice led to differences in whole-bone mechanical properties. On examination by four-point bending tests at the cortical midshaft, HIT mice showed an increase in maximum load at both 8 and 16 weeks and a significant increase in stiffness by 16 weeks (Fig. 4C, D), as expected from the increased moment of inertia and TMD.

**Fig. 5:**
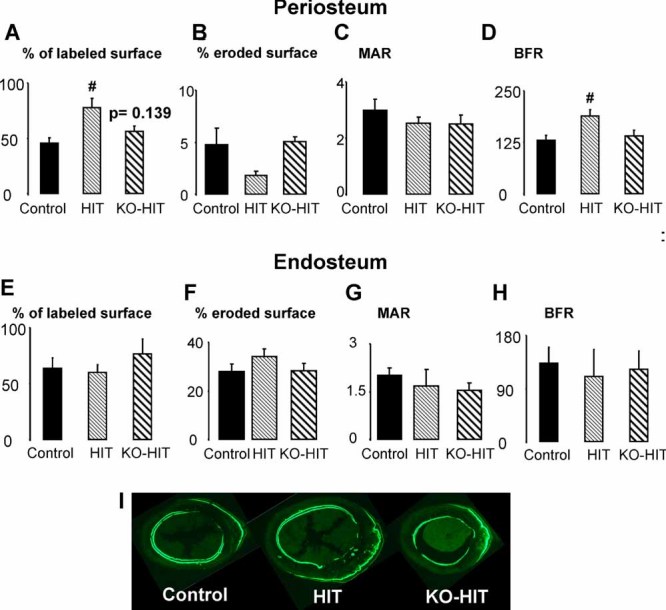
Elevated levels of serum IGF-1 increase periosteal bone-formation rate and labeled perimeter only in the presence of local igf1 gene expression. Cortical bone histomorphometry was assessed from 30-µm plastic sections of the femoral midshaft. Percent labeled surface, percent eroded surface, mineral apposition rate (MAR), and bone-formation rate (BFR) were measured at the periosteal (A–D) and endosteal (E–H) surfaces. Cross-sectional images (I) were acquired at ×4 magnification and are representative sections of each mouse strain (n = 5 mice per group).

To understand the cellular mechanism that led to the HIT phenotype, we determined serum levels of ostocalcin, a bone-formation marker, and performed histomorphometric analysis at the femoral midshaft. Serum osteocalcin at 8 weeks of age increased significantly in HIT mice compared with controls (Fig. 4E). Accordingly, we found that in HIT mice, percent labeled surface at the periosteum increased significantly (Fig. 5). Additionally, although mineral apposition rate (MAR) did not differ significantly between control and HIT mice on the endosteal or periosteal surfaces, periosteal bone formation rate (BFR) increased significantly (Fig. 5), which was in accordance with increased total cross-sectional area, as shown by µCT analyses.

### When autocrine/paracrine IGF-1 expression is absent, elevated serum IGF-1 fails to prevent early deficiencies but restores cortical bone morphology and mechanical properties after puberty

Despite threefold elevation in serum IGF-1 above control levels, KO-HIT mice exhibited significantly shorter femurs (–4.7% decrease), reduced Tt.Ar (–14.2% decrease) and cortical area (Ct.Ar) (–14.3% decrease), and a more slender, less robust phenotype (reduced Tt.Ar/Le) at 4 weeks of age (Fig. 2). However, between 4 and 8 weeks, KO-HIT mice exhibit marked increase in growth rate, resulting in increases in Tt.Ar (+5.4-fold) and Ct.Ar (+1.9-fold) above controls (Fig. 4A, B). During pubertal growth (4 to 8 weeks), KO-HIT mice underwent a marked subperiosteal expansion such that by 8 weeks their morphologic properties equaled or surpassed those of controls. In addition, while the mechanical properties of KO-HIT mice were similar to controls at 8 weeks, by 16 weeks of age, increases in Ct.Ar and Ct.Th produced a striking 24.6% increase in maximum load (*p* < .01) and a 12.5% increase in stiffness (n.s.) above control values (Fig. 4C, D). In trabecular bone, none of the morphologic features examined in KO-HIT mice at either 8 or 16 weeks differed from controls despite diminished autocrine/paracrine IGF-1 expression and increased serum IGF-1 levels (Fig. 3).

Unlike HIT mice, serum osteocalcin (Fig. 4E) in KO-HIT mice decreased when compared with controls. This may indicate that a secondary factor, regulated by tissue-IGF-1, is required for enhancement of osteocalcin secretion by osteoblasts. Interestingly, despite similar elevations in serum IGF-1 between HIT and KO-HIT mice, we found that all parameters of bone formation or resorption on both periosteal and endosteal surfaces in KO-HIT mice were similar to controls. Moreover, assessment of osteoblast and osteoclast number and distribution along the periosteal and endosteal surfaces did not differ among the groups (Table 1).

**Table 1:** Number of Osteoblasts and Osteoclasts Along the Periosteal and Endosteal Surfaces of the Mid-diaphysis of the Femur (number of cells/mm)

	Endosteum		Periosteum	
		
	Osteoblasts	Osteoclasts	Osteoblasts	Osteoclasts
Control	8.60 ± 2.22	7.32 ± 2.49	5.85 ± 1.12	3.53 ± 2.17
HIT	6.16 ± 2.12	7.09 ± 1.89	2.69 ± 0.99	2.91 ± 0.63
KO-HIT	4.79 ± 1.29	4.47 ± 1.04	3.17 ± 1.33	6.54 ± 1.22

## Discussion

**Fig. 6:**
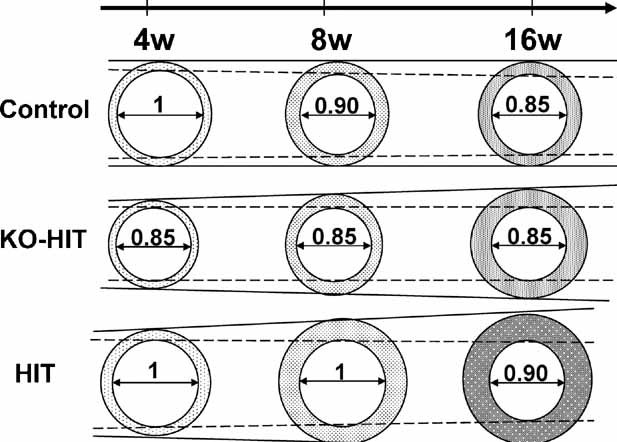
Schematic illustration of bone-growth patterns in mice with altered tissue and/or serum IGF-1 levels. Female control mice show a slight increase in total cross-sectional area during the first 8 weeks of life. Later on, a marked increase in cortical bone area and TMD, associated with marrow in-filling, allows an efficient structure to support the age-related increase in body weight. KO-HIT female mice exhibit a significantly smaller total cross-sectional area at 4 weeks, but at 8 weeks they show a “catch up” growth and do not differ significantly from controls. Nonetheless, between 8 and 16 weeks, KO-HIT female show a significant increase in total cross-sectional area associated with a marked increase in cortical thickness and no changes in marrow area, suggesting increased periosteal bone apposition. In contrast, HIT mice show significant increases in both total cross-sectional area and cortical bone area between 4 and 8 and 8 and 16 weeks of age. These increases were associated with a decrease in marrow area, suggesting that both periosteal and endosteal bone apposition took place.

The results of this study demonstrate clear differences in the roles played by circulating (liver-derived, endocrine) IGF-1 and locally produced (autocrine/paracrine) IGF-1 in skeletal development. In particular, autocrine/paracrine IGF-1 appears essential to establish skeletal features during early development (before 4 weeks) because overexpression of circulating (liver) IGF-1 in the absence of normal autocrine/paracrine IGF-1 production failed to prevent low body length and early skeletal deficits. On the other hand, overproduction of circulating IGF-1 by liver fully restored these deficits during late development such that KO-HIT mice exhibited “catch-up” growth and skeletal properties at 8 and 16 weeks that were equal to control mice (Fig. 6). Collectively, the data derived from the skeletal characterization of the KO-HIT mice suggest that in the absence of tissue-derived IGF-1 and in the presence of excess serum IGF-1 (that redistributes in tissues), skeletal growth and acquisition are normalized. Also of interest, the effects of endocrine and autocrine/paracrine IGF-1 were independent of changes in GH, which remained unaltered from control levels in both HIT and KO-HIT mice during the entire experiment. This contrasts with our previous results in LID mice, where ablation of liver IGF-1 production produced a variety of skeletal effects, but these were superimposed on elevated GH levels.(10)

The postnatal effects of elevated circulating IGF-1 in HIT and KO-HIT mice appear consistent with IGF-1 actions in humans, where fetus serum levels of IGF-1 are relatively low (∼30% of adult levels),(26) and a gradual rise is seen during childhood, with attainment of adult levels at the onset of puberty. However, during puberty, serum IGF-1 concentrations rise further by three- to fourfold and then decline gradually between 20 and 30 years of age. The concentrations of IGF-1 in serum during puberty correlate better with “bone age” than with chronologic age.(27) Interestingly, our study shows that elevations in serum IGF-1 levels during postnatal growth correlate with enhanced morphologic and mechanical properties of bone at the end of puberty (∼16 weeks). This resembles human skeletal growth, where serum IGF-1 elevates three- to fourfold during puberty and augments bone growth.(28)

In a recent study using a knock-in genetic approach, Stratikopoulos and colleagues showed that endocrine IGF-1 accounts for approximately 30% of overall body growth.(29) In that study, knock-in of the igf1 gene exclusively in liver of igf1 null mice partially restored serum IGF-1 levels to 44% of control, resulting in body weights that were approximately 50% of controls compared with 30% in igf1 null animals.(29) Skeletal growth was not assessed in detail in that study, nor were bone properties examined; however, based on the relationships between body weight and bone properties observed in this study and elsewhere,(10,30) it appears likely that those adult skeletons also would have diminished features relative to normal controls. Thus, while our studies indicate that elevated levels of circulating IGF-1 can restore normal postnatal skeletal development, whether normal IGF-1 levels would suffice has yet to be established.

Our results showing how the phenotypic characteristics of the bones in HIT and KO-HIT mice evolved from 4 to16 weeks postnatally confirmed previous findings by Jing and colleagues in which overexpression of IGF-1 in osteoblasts, which resulted in concomitant increases in serum IGF-1, led to increases in bone length, Ct.Th, Ct.Ar, and Tt.Ar.(31) In our study, we also found that cortical bone thickness in HIT mice increased gradually from 4 to 16 weeks and was accompanied by decreases in marrow area, suggesting that both endosteal and periosteal bone apposition took place. In contrast, despite similar elevations in serum IGF-1 levels, KO-HIT mice were shorter with a smaller Tt.Ar than HIT and control mice at 4 weeks of age. In our previous study, using LID mice (which Exhibit 75% reductions in serum IGF-1 levels), we showed that endocrine IGF-1 deficiency resulted in a slender bone phenotype(10) affecting mostly cortical bone and with no adverse effects on cancellous bone. The slender phenotype of LID mice (reduced Tt.Ar/Le) appeared after 4 weeks of age, when serum IGF-1 levels peak naturally. In this study, we also found that endocrine IGF-1 regulates mostly cortical bone architecture and does not have major effects on cancellous bone. However, we demonstrated that unlike male LID mice, female HIT mice, with elevated serum levels of IGF-1, display a robust bone phenotype (increased Tt.Ar/Le) that is independent of body weight (data not shown) and that indices of robustness (eg, Tt.Ar/Le, Tt.Ar, and Ct.Ar) were significantly increased at 4 weeks of age. Interestingly, when tissue IGF-1 action was ablated (KO-HIT), elevated levels of serum IGF-1 were able to restore linear growth and skeletal morphology after 4 weeks of age by virtue of accelerated growth rates. This implies that additional IGF-1-dependent local factors are necessary to promote skeletal growth during the neonatal and prepubertal growth phase. Moreover, despite the fact that skeletal morphology was restored to control levels as a result of “catch up” growth in the KO-HIT mice by 8 weeks of age, we did not detect further increases in Tt.Ar, TMD, or robustness by 16 weeks, suggesting that both endocrine and autocrine/paracrine functions are required for establishment of a robust bone phenotype as seen in the HIT mice. Interestingly, in KO-HIT mice, we found no changes in marrow area from 4 to 16 weeks of age, whereas cortical thickness and total area increased owing to periosteal bone apposition. These data are in accordance with our previous finding in LID mice (with decreased serum IGF-1 levels), where periosteal bone apposition was inhibited by 8 weeks of age, and a compensatory mechanism of marrow in-filling was evident at 32 weeks of age.(10) Dynamic histomorphometric measurements of HIT bones showed an increase in bone-formation rate on the periosteal surface, which was in accordance with increased serum osteocalcin levels and increased Tt.Ar measured by µCT. Following bone growth in HIT mice from 4 to 16 weeks of age, we found increases in Tt.Ar, Ct.Ar, and Ct.Th, accompanied by decreases in Mr.Ar. Together, the µCT and histomorphometric data suggest that processes of bone resorption and apposition on the periosteal and endosteal surfaces were coupled in HIT mice. In contrast, in KO-HIT mice, despite similar elevations in serum IGF-1 levels, osteocalcin levels in serum were reduced, but all indices of bone formation and resorption, measured by histomorphometry, were indistinguishable from controls. µCT analysis showed increases in KO-HIT Ct.Ar during growth, whereas no changes in Mr.Ar were observed. Collectively, these data may indicate uncoupling of bone resorption and apposition on the periosteal and endosteal surfaces and suggest that secondary factor(s), regulated by tissue IGF-1, may be required for the establishment of bone-growth and bone-drift processes during normal development. Thus elevations of IGF-1 in serum can compensate for the absence of tissue IGF-1 during postnatal growth, but tissue IGF-1 is obligatory for development of the neonatal skeleton.

IGF-1 plays an important role in bone mineralization, as evidenced by osteoblast-specific IGF-1 receptor gene ablation(32) impairing coupling of matrix biosynthesis and mineralization and by mice overexpressing IGF-1 specifically in osteoblasts, which show increases in BMD as early as 6 weeks of age.(33) Similarly, in LID mice, with reduced serum IGF-1 levels, mineralization lag time increased threefold over controls.(10) In this study we found that increases in serum IGF-1 levels lead to increased TMD in HIT mice and, consequently, greater stiffness and maximum load values. However, this increase was not detected in the KO-HIT mice, suggesting that both tissue IGF-1-dependent factors and serum IGF-1 are required for complete regulation of mineralization. Moreover, in the absence of tissue IGF-1, dependence of mechanical function on morphology may be increased. In support of this, KO-HIT mice exhibit an increase in maximum load likely owing to increased Ct.Ar, Ct.Th, and RCA because TMD was not changed.

Long-term treatment (2 to 7 years) with recombinant human (rh)IGF-1 in prepubertal children(13–15,34) produces accelerated linear growth, with the highest growth velocity achieved within the first year of treatment. In a few cases, a fast “catch up” growth of head circumference was reported as well as progressive growth of the extremities (hands, feet, chin, and nose). Data reported at the 2004 Annual Meeting of the Endocrine Society demonstrated statistically significant increases in both linear growth and growth rate over an 8-year period in response to rhIGF-1 therapy. Compared with pretreatment growth patterns, on average, children gained an additional inch per year for each year of therapy over the course of 8 years. Our current results are in agreement with those findings, in that elevated serum IGF-1 levels during development restore linear growth and do not cause metabolic abnormalities. Moreover, in contrast to human studies in which bone properties cannot normally be examined in detail, our findings reveal compartment-specific effects of IGF-1 on the skeleton (in particular, the cortical shell) and also demonstrate directly the functional consequences of elevated serum IGF-1 levels on mechanical properties of bone.

Finally, it is important to note that in recent years IGF-1 has been linked with several types of malignancy. While cause-effect relationships have not been established, caution is routinely exercised when treating children with IGF-1. Our study only examined mice into adulthood, and while we observed no obvious pathologic consequences in these animals, longer-term studies may be needed to address the possibility that high IGF-1 levels during growth affect tumor development or other pathology later in life.
